# The Prevalence of the Virulence Genes of *Staphylococcus aureus* in Sickle Cell Disease Patients at KSUMC, Riyadh, Saudi Arabia

**DOI:** 10.3390/antibiotics12071221

**Published:** 2023-07-22

**Authors:** Adel A. Abdulmanea, Naiyf S. Alharbi, Ali M. Somily, Jamal M. Khaled, Farjah H. Algahtani

**Affiliations:** 1 Department of Botany and Microbiology, College of Science, King Saud University, P.O. Box 2455, Riyadh 11451, Saudi Arabia; nalharbi1@ksu.edu.sa (N.S.A.); gkhaled@ksu.edu.sa (J.M.K.); 2 Department of Pathology, College of Medicine, King Saud University and King Saud University Medical City, P.O. Box 2925, Riyadh 11451, Saudi Arabia; somily@ksu.edu.sa; 3 Department of Hematology, College of Medicine, King Saud University and King Saud University Medical City, P.O. Box 2925, Riyadh 11451, Saudi Arabia; falgahtani@ksu.edu.sa

**Keywords:** sickle cell disease, Saudi Arabia, virulence factors, antibiotics, antimicrobial resistance, MRSA

## Abstract

*Staphylococcus aureus* in the blood of sickle cell disease (SCD) patients may result in a significant increase in morbidity and mortality. *S. aureus* strains contain various virulence characteristics, including the ability to create a variety of toxins and develop drug resistance. The current study sought to assess the prevalence of *S. aureus* in SCD patients and to identify the pathogen’s virulence characteristics. Between 2017 and 2021, blood samples and data were collected at King Saud University Medical City (KSUMC) in Riyadh, Saudi Arabia. The Vitek system PCR and gene sequencing methods were used for identification, antibiotic resistance patterns, and genetic analysis. During the study period, 47 *S. aureus* blood isolates (methicillin-resistant *S. aureus* (MRSA) 41.6% and non-MRSA 58.4%) were isolated from 2406 SCD patients. The prevalence percentages of virulence genes (*finbB*, *sdrC*, *sdrD*, *icaA*, *coa*, *nuc*, *hlg*, *hla*, *finbA*, *clfA*, *efb*, *pvl*, *agr*, *spa*, *seb*, *sea*, *sec*, *tst*, and *sed*) among all the isolates from the SCD patients compared with non-SCD patients (control group) were as follows: (100% vs. 100%), (100% vs. 100%), (100% vs. 100%), (100% vs. 87.5%), (100% vs. 81.3%), (100% vs. 100%), (100% vs. 100%), (100% vs. 100%), (97.9% vs. 81.3%), (97.9% vs. 100%), (97.9% vs. 87.5%), (54.3% vs. 56.3%), (46.8% vs. 75%), (42.6% vs. 43.8%), (27.7% vs. 0%), (25.5% vs. 12.5%), (12.8% vs. 6.3%), (4.3% vs. 12.5%), and (4.3% vs. 0%). Regarding the resistance genes (*plaZ*, *mecA*, *ermA*, *ermC*, *tetK*, *tetM*, and *ermB*) of the *S. aureus* strains isolated from the SCD patients compared with non-SCD patients (control group), the prevalence percentages were as follows: (100% vs. 100%), (100% vs. 56.3%), (0% vs. 31.3%), (31.9% vs. 18.8%), (40.4% vs. 25%), (0% vs. 0%), and (0% vs. 0%). As for the antibiotic (ampicillin, penicillin, amoxicillin, cefazolin, imipenem, oxacillin, erythromycin, tetracycline, azithromycin, ciprofloxacin, moxifloxacin, and levofloxacin) resistance of the *S. aureus* strains isolated from the SCD patients compared with non-SCD patients (control group), the prevalence percentages were as follows: (100% vs. 100%), (97.9% vs. 100%), (72.3% vs. 25%), (68.1% vs. 37.5%), (68.1% vs. 25%), (66% vs. 25%), (36.2% vs. 18.8%), (23.4% vs. 12.5%), (19.1% vs. 12.5%), (17% vs. 12.5%), (14.9% vs. 25%), and (10.6% vs. 18.7%). This study concluded that several virulence genes were present in the *S. aureus* strains recovered from the SCD patients at KSUMC, with all the isolates containing the *finbB*, *sdrC*, *sdrD*, *icaA*, *coa*, *nuc*, *hlg*, and *hla* genes.

## 1. Introduction

*Staphylococcus aureus* is considered to be an opportunistic pathogen and common cause of bloodstream infections, associated with high mortality and morbidity in most hospitals [1]. In addition, *S. aureus*, especially methicillin-resistant *S. aureus* (MRSA), is a common cause of nosocomial infections and is considered to be a major problem for public health [2]. It is a Gram-positive bacterium that causes infections, such as pneumonia, septicemia, endocarditis, osteomyelitis, meningitis, and toxic shock syndrome, with high levels of antimicrobial resistance [3,4]. MRSA infections are increasing worldwide with a high mortality and morbidity, and since it has multidrug resistance, there are narrow therapeutic options, which makes it a significant health concern [5]. The prevalence of MRSA bacteria in Saudi Arabia is 50% in patients [6]. *S. aureus* produces many virulence factors, such as enterotoxins, exoenzymes, immune-modulatory factors, hemolysins, leukocidins, proteases, and exfoliative toxins, which allow it to adhere to a surface, invade or avoid the immune system, and, in addition, cause toxicity to the host [7].

Sickle cell disease (SCD) is a comprehensive term that refers to a group of genetic disorders associated with structurally abnormal hemoglobin. It occurs when an individual inherits a sickle (*HbS*) gene from one parent and another abnormal beta-globin gene, often sickle hemoglobin (*HbS*), from the other parent. The most common type of SCD is homozygous SCD (HbSS or ss), also known as sickle cell anemia. Other less common but clinically important forms of SCD include sickle cell-beta-thalassemia (HbS/β-thalassemia) and sickle cell-HbC (HbSC or HbS/C) disease [8]. Globally, about 5% of the world’s population carries the genes responsible for hemoglobinopathies. SCD affects more than 250,000 live births worldwide per year [9]. Sickle cell anemia is one of the most common inherited disorders in Saudi Arabia [10]. Patients with SCD are known to have an increased risk of invasive bacterial infections (IBI), resulting in frequent hospitalization and the intake of antibiotic treatment. Some researchers studying SCD have usually focused on the occurrence of encapsulated bacterial pathogens, such as *Streptococcus pneumoniae*, *Haemophiles influenzae*, *Neisseria meningitis*, *Salmonellae* spp., and *Klebsiella* spp., in SCD patients [11,12]. Yet, among SCD patients, *S. aureus* has a higher potential for causing invasive diseases than *S. pneumonia* [11].

Over the last few decades, multidrug-resistant strains of *S. aureus* have emerged worldwide as a result of the widespread use of antibiotics. The emergence of resistance in *S. aureus* is attributed to various mechanisms, including the production of enzymes that inactivate antimicrobial agents, the activation of antimicrobial efflux pumps, the reduction in bacterial cell permeability to antibiotics, and the modification of the target site for the antibiotic [13]. The emergence of drug-resistant and virulent strains of *S. aureus*, specifically MRSA, poses a serious challenge to the treatment and management of staphylococcal infections. These methicillin-resistant strains cause difficult-to-treat infections because they are resistant to several antibiotics, including beta-lactams, aminoglycosides, and macrolides. The primary mechanism of penicillin resistance is the production of beta-lactamase, which inactivates penicillin by breaking down its beta-lactam ring. Another mechanism associated with the presence of penicillin-binding protein 2a (PBP2a) is encoded by the *mecA* gene, which is carried on the staphylococcal cassette chromosome mec (SCCmec). Additionally, the gene *blaZ* is involved in penicillin resistance in staphylococci and encodes for the production of β-lactamase [14]. However, the relationship between antibiotic resistance and individuals with SCD has not been adequately studied. Few studies have reported on *S. aureus* and MRSA bacteria in SCD patients in Saudi Arabia. Therefore, large prospective studies are needed to determine the epidemiology of these bacteria in patients with SCD, including carriage rates, the determinants of carriage, the antibiotic resistance of colonizing strains, and virulence factors, in order to detect specific risk factors.

Our study is the first in Saudi Arabia to indicate the presence of several virulence factors and antibiotic resistance genes in clinical *S. aureus* and MRSA isolates among SCD patients. In the present investigation, PCR was utilized to identify the bacterial strains (N = 47) obtained through sequencing of the 16S rRNA gene. For each isolate, the virulence genes (*clfA*, *fnbA*, *fnbB*, *sdrC*, *sdrD*, *spa*, *sea*, *seb*, *sec*, *sed*, *hla*, *hlg*, *pvl and tst*, *coa*, *efb*, *icaA*, *nuc* and *agr*) and the genes responsible for multiple antibiotic resistance (*plaZ*, *mecA*, *ermA*, *ermB*, *ermC tetM*, and *tetK*) were examined.

The main contribution of the present work is the analysis of the antibiotic susceptibility and genetic patterns for *S. aureus* strains isolated from SCD patients who had a bloodstream infection. This investigation’s findings could help researchers to better understand the nature of these deadly bacteria that endanger the lives of those suffering from SCD.

## 2. Materials and Methods

### 2.1. Study Ethics

This study was performed at the Botany and Microbiology Department, College of Science, King Saud University (KSU), and Microbiology Department, KSU, Medical City (KSUMC). All procedures and protocols, including the sample collection, bacteria isolation, and data analysis, were approved by the institutional review board (IRB) at KSUMC, Riyadh, Saudi Arabia (Institutional Review Board No. 18/0258/IRB 12 April 2018).

### 2.2. Study Design and Sample Size

The collection and preparation of the samples were carried out at KSUMC, Faculty of Medicine, Department of Medical Microbiology, between January 2017 and December 2021. In total, 30 and 17 isolates of MRSA and non-MRSA, respectively, were isolated from 2406 SCD patients (HbSS), and 16 strains of *S. aureus* without the SCD disease (control group), from patients ranging in age from 1 to 63 years old. After obtaining the appropriate institutional review, the charts of the patients with SCD (HbSS) and bloodstream infections were reviewed for those who had visited the hospital (KSUMC) for five years (2017–2021). The clinical isolates (N = 47) of *S. aureus* and MRSA were isolated from blood. All the isolates were obtained from the laboratory of microbiology at KSUMC from the SCD patients and had positive blood cultures with *S. aureus* infection. The isolates were stored in skim milk medium with 10% glycerol at −80 ± 5 °C. The total DNA of each pure isolate was extracted and then transported to the Microbiology Laboratory, Botany and Microbiology Department, College of Science, KSU for further testing and a genetic analysis.

### 2.3. Bacterial Isolation and Identification

The identification of the isolated bacteria was carried out using conventional methods, including Gram stain, morphological, biochemical tests, VITEK 2 C15 configuration Automated system (Biomérieux, Marcy-l'Étoile, France), and serology tests; then, the molecular method (PCR assay) was used to confirm this identification. After the DNA was extracted with a DNeasy1 Blood & Tissue Kit (Qiagen, Hilden, Germany), the 16S rRNA genes were targeted to confirm *S. aureus*. The commercially available master mix (2x HotStarTaq Plus Master Mix; Qiagen, Hilden, Germany) was used to set up the PCR reaction. The reaction mixture used in this study included: 5 μL of DNA, 20 μL of PCR mixture of 2x HotStarTaq Plus Master Mix (Qiagen, Hilden, Germany), and 1.5 μM of each forward and reverse primer. Target gene amplification was performed using PCR with a Thermal Cycler Corbett Research PCR Thermal Cycler. The thermal cycling conditions were optimized with an initial denaturation at 95 °C for 15 min, 35 cycles (denaturation, 94 °C, 1 min; annealing, 54 °C, 1 min; extension, 72 °C, 1 min), with a final extension at 72 °C for 10 min, and held at 4 °C.

### 2.4. Antibiotic Susceptibility Testing

The susceptibility of the *S. aureus* isolates to different antimicrobial agents was tested by using an automated susceptibility testing machine VITEK 2 system (Biomerieux) [15]. An AST-GP67Test Kit (bioMerieux, Inc., Durham, NC, USA), Gram-positive susceptibility card was used to determine the sensitive, intermediate, and resistant isolates against 26 various antibiotics including (amoxicillin/clavulanic acid (Amox), ampicillin (Amp), azithromycin (Azith), cifazolin (Cefaz) ciprofloxacin (Cipro), clindamycin (Clinda), daptomycin (Dapto), erythromycin (Eryth), fosfomycin (Fosfo), fusidic acid (FusA), gentamicin (Genta), imipenem (Imi), levofloxacin (Levo), linezolid (Lzd), moxifloxacin (Mox), mupirocin (Mup), rifampin (Rif), penicillin (Pen), oxacillin (Ox), tetracycline (Tetra), tobramycin (Tobra), trimethoprim/sulfamethoxazole (Sxt), synercid (Synercid), tigecycline (Tgc), teicoplanin (Teico), and vancomycin (Vanc) according to the Clinical Laboratory Standards Institute’s (CLSI) recommendations. The VITEK 2 is an automated microbiology system utilizing growth-based technology. Antibiotic susceptibility patterns were analyzed for *S. aureus* strains (case) isolated from the patients with SCD (HbSS) (N = 47) and *S. aureus* strains (control) isolated from the individuals without SCD (N = 16).

### 2.5. DNA Extraction and PCR

#### 2.5.1. DNA Extraction

The DNA of all the *S. aureus* isolates was extracted using DNeasy Blood & Tissue Kits (Qiagen, Germantown, MD, USA), following the manufacturer’s instructions, as per the following:

1. In total, 3 ml of growth cultures was centrifuged and the pellets were washed twice with a sterile saline solution 0.89% Nacl. The supernatant was discarded and the pellet was re-suspended in 180 µL of enzymatic lysis buffer and incubated for at least 30 min at 37 °C.
2. Then, 25 µL of Proteinase K and 200 µL of Buffer AL (without ethanol) were added and mixed by vortex for 15 s and incubated at 56 °C for 30 min. A total of 200 µL of ethanol (96%) was added and mixed by vortexing.
3. After that, the mixture was put into the DNeasy Mini spin column with a 2 mL collection tube and centrifugation was performed at 6000× *g* (8000 rpm) for 1 min.
4. The spine column was placed in a new 2 mL collection tube, and 500 µL of WA1 buffer was added and centrifuged at 6000× *g* (8000 rpm) for 1 min.
5. As in the previous step, the spin column was placed again in another 2 mL collection tube, and 500 µL of AW2 buffer was added and centrifuged at 20,000× *g* (14,000 rpm) for 3 min.
6. Finally, the spine column was placed in a clean 2 mL microcentrifuge tube and 100 µL of buffer elution (AE) was added and centrifuged at 6000× *g* (8000 rpm) for 1 min to elute the DNA. The eluted DNA was stored at −75 °C until use.

#### 2.5.2. Detection of Virulence Factors Genes of *S. aureus*

Polymerase chain reaction (Corbett Research PCR Thermal Cycler) was used for the determination of the 16s RNA region for the genus, as well as nineteen virulence genes, including, adhesion-associated genes (*clfA*, *fnbA*, *fnbB*, *sdrC*, *sdrD*, and *spa*), enterotoxin A, B, C, and D genes (*sea*, *seb*, *sec*, and *sed*), other exotoxin genes (*hla*, *hlg*, *pvl*, and *tst*), and other (*coa*, *efb*, *icaA*, *nuc*, and *agr*) genes, by using the list of specific primers shown in Table 1. The DNA of the *S.aureus* isolates was extracted using the DNeasy Blood & Tissue Kits (Qiagen, Germantown, MD, USA). Nineteen virulence genes were detected by multiple-PCR using the appropriate specific primers and electrophoresed amplicons on a 1–2% agarose gel with 0.5 μg/mL of ethidium bromide stain. The primer genes were selected based on earlier studies [16,17].

#### 2.5.3. Determination of Antibiotic Resistance Genes of *S. aureus*

The bacterial DNA was extracted using DNeasy Blood & Tissue Kits (Qiagen, Germantown, MD, USA). Polymerase chain reaction (PCR) was used for the detection of various antibiotic resistance determinants in the isolated clinical *S. aureus* (N = 47), according to previously reported methods [14]. Resistance to tetracycline was detected by the presence of the *tetK* and *tetM* genes. Erythromycin resistance was detected by the presence of the *ermA*, *ermB*, and *ermC* genes. Resistance to penicillin and oxacillin was detected by the presence of the *blaZ* and *mecA* genes, respectively. Amplification was carried out via a PCR amplification of various antibiotics resistance genes by using the list of specific primers shown in Table 2. The DNA amplification was carried out in a PCR thermocycler (Corbett Research PCR Thermal Cycler), with the following reaction conditions: initial denaturation for 15 min at 95 °C, followed by 35 cycles of denaturation for 1 min at 94 °C, 30 s at annealing temperature of each primer, extension at 72 °C for 1 min, and a final extension for 10 min at 72 °C. The amplified genes were analyzed using 1.5–2% agarose gel electrophoresis. The primer genes were selected based on earlier studies [14].

### 2.6. Statistical Analysis

The data from the study will be entered initially into the Microsoft Office Excel program, then into the Statistical Package for the Social Sciences (SPSS) program. The data were first summarized in the form of frequencies and percentages and then subjected to a series of statistical analyses. Independent samples, Chi-square, and binary logistic regression were used to compare the data from the different groups among the study participants. Simple statistical tools, such as descriptive statistics and proportion t-tests, were used to test for significance. *p* value (*p* < 0.05) and the power of the test set was at 0.8, with test proportion 0.01.

## 3. Results

### 3.1. Incidence of S. aureus Strains among Patients with SCD

During the 5-year study period, 2406 patients were diagnosed with SCD, 138 patients had bloodstream infections (5.7%) with some types of bacteria, 17 patients had *S. aureus* (9 male and 8 female), and 30 patients had MRSA (21 male and 9 female). The percentage of *S. aureus* and MRSA in the SCD patients was (2%).

The age groups were divided according to the high-risk population for infection with SCA. There were two segregated groups, such as ≤20 years and >20 years. The most affected age group was (>20 years) of age, with (61.7%) of the total number of samples.

### 3.2. Identification of S. aureus Isolates

#### 3.2.1. Identification of *S. aureus*

The findings indicated that *S. aureus* is characterized by specific cultural, phenotypic, and biochemical features, including mannitol fermentation, golden-yellow colony formation, catalase positivity, oxidase positivity, and coagulase positivity.

#### 3.2.2. Identification of Isolates by 16S rRNA Gene Sequence

The results (See Figure S1 in Supplementary Data) indicated that the genomic DNA was successfully purified from the different isolates with no DNA shearing or impurities. The results indicated that the 16S rDNA of the different isolates were successfully amplified, with the correct size of about 1525 bp. The data of the 16S-rDNA sequence obtained from the GeneBank database confirmed that all the isolated strains (100%) had been identified as *S. aureus* strains and that the similarity of the BLAST analysis ranged between 96 and 99%.

### 3.3. Antibiotic Susceptibility Testing

**Figure 1:**
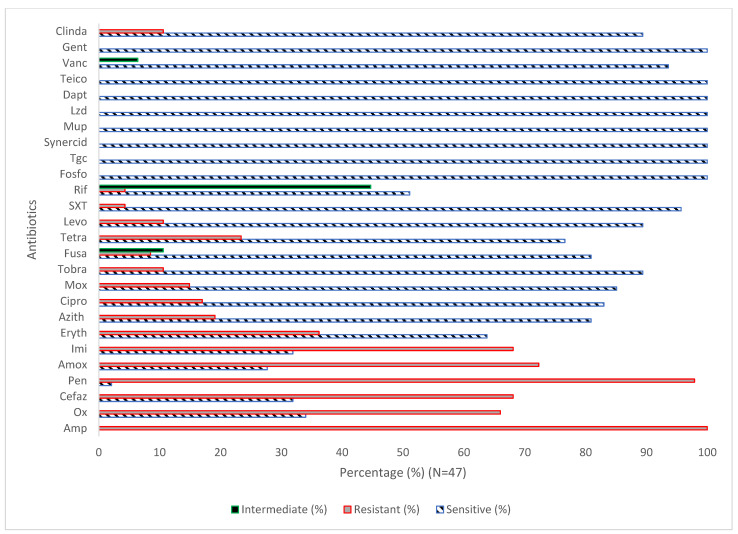
Antibiotic susceptibility pattern of *S. aureus* isolates (N = 47). Amp, ampicillin; Ox, oxacillin; Cifaz, cefazolin; pen, penicillin; Amox, amoxicillin Imi, imipenem; Eryth, erythromycin; Azith, azithromycin; Cipro, ciprofloxacin; Mox, moxifloxacin; Tobra, tobramycin; Fusa, fusidic acid; Tetra, tetracycline; Levo, levofloxacin; SXT, trimethoprim-sulfamethoxazole; Rif, rifampin; Fosfo, Fosfomycin; Tgc, tigecycline; Synercid, synercid; Mup, mupirocin; Lzd, linezolid; Dapt, daptomycin; Teico, teicoplanin; Vanc, vancomycin; Gent, gentamicin; and Clinda, clindamycin.

The susceptibility testing of the *S. aureus* strains (N = 47) toward various antibiotics (N = 26) showed that 100%, 97.9%, 72.3%, 68.1%, 68.1%, 66%, 36.2%, 23.4%, 19.1%, 17%, 12.8%, 14.9%, 10.6%, 10.6%, 6.4%, 8.5%, 4.3%, and 4.3% of the isolates were resistant to Amp, Pen, Amox, Cefaz, Imi, Ox, Eryth, Tetra, Azith, Cipro, Mox, tobra, levo, fusa, SXT, and Rif, respectively (Figure 1). The data revealed that 100% of the isolates were sensitive to Fosfo, Tgc, Synercid, Mup, Lzd, Dapt, Teico, and Van. In addition, the findings showed that 10.6%, 44.7%, and 6.4 of the isolates were intermediate to Fuso, Rif, and Vanc, respectively. The results showed significant differences (*p* < 0.05) between the case and control groups for Amox, Cefaz, Ox, and Rif Table 3 and Table 4. The results, summarized in Table 5, revealed significant variation in the antibiotic susceptibility among MRSA (N = 30) and *S. aureus* (N = 17).

### 3.4. Detection of Virulence Genes of S. aureus Isolates

We investigated the presence of 19 virulence genes responsible for the pathogenicity of the isolated S. aureus (N = 47), using a set of PCR primers specific to various virulence genes, including adhesion-associated genes (*clfA*, *fnbA*, *fnbB*, *sdrC*, *sdrD*, and *spa*), enterotoxin genes (*sea*, *seb*, *sec*, and *sed*), other exotoxin genes (*hla*, *hlg*, *pvl*, and *tst*), and others (*coa*, *efb*, *icaA*, *nuc*, and *agr*)”.

**Figure 2:**
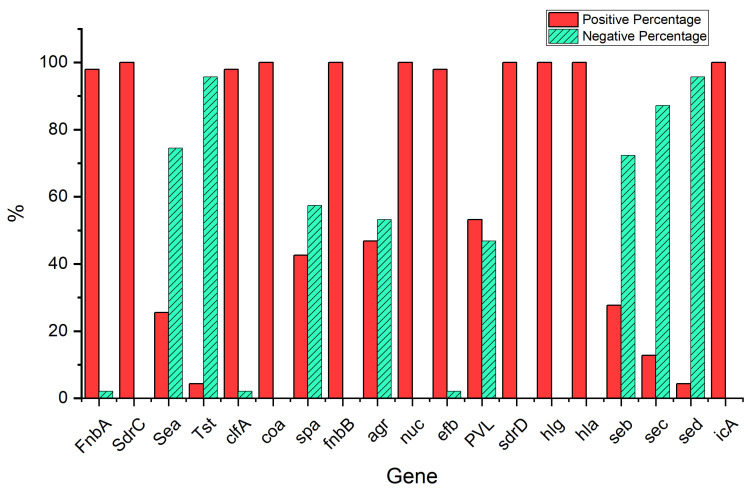
Prevalence of virulence genes of *S. aureus* isolates (N = 47) patients.

As observed in Figure 2, of the *S. aureus* isolates obtained from the patients with SCD, the current study confirmed the finbA gene carriage in 97.9% (N = 46), and the *sdrC*, *sea*, *tst*, *clfA*, *coa*, *spa*, *finbB*, *agr*, *nuc*, *efb*, *pvl*, *sdrD*, *hlg*, *hla*, *seb*, *sec*, *sed*, and *icaA* genes were demonstrated in 100% (N = 47), 25.5% (N = 12), 4.3% (N = 2), 97.9% (N = 46), 100% (N = 47), 42.6% (N = 20), 100% (N = 47), 46.8% (N = 22), 100% (N = 47), 97.9% (N = 46), 54.3% (N = 25), 100% (N = 47), 100% (N = 47), 100% (N = 47), 27.7% (N = 13), 12.8% (N = 6), 4.3% (N = 2), and 100% (N = 47), respectively. The results showed that there were no significant differences (*p* < 0.05) in the prevalence of positive virulence genes among the *S. aureus* isolates (N = 47) between the MRSA (N = 30) and non-MRSA (N = 17) isolates, nor between males (N = 30) and females (N = 17) Table 6, Table 7 and Table 8 indicate variation in the presence of virulence genes between the case, control, MRSA, and non-MRSA isolates. Moreover, Table 9 and Table 10 show variation in the proportions of both virulence genes and antibiotic resistance. The results (See Figure S2 in Supplementary Data) indicate that the virulence genes used in this study were successfully amplified with the correct size for the different isolates.

### 3.5. Determination of Antibiotic Resistance Genes of S. aureus Strains

The results of the PCR assay indicted that the highest resistance genes were obtained against β-lactam antibiotics, blaZ (100%) and mecA (100%) in penicillin and oxacillin in the *S. aureus* isolates. Overall, the antibiotic resistance gene rates for tetracycline and erythromycin were 40.4 and 31.9 percent, respectively. The results indicate (See Figure S3 in Supplementary Data) that the antibiotic resistance genes used in this study were successfully amplified with the correct size for the different isolates. The results showed significant differences (*p* < 0.05) between the case and control groups in the *mecA*, *ermA*, and *ermC* genes Table 10. The results, summarized in Table 11, reveal significant variation in the *tetK* and ermC genes among the MRSA (N = 30) and *S. aureus* (N = 17) isolates. The results of the PCR products analysis (See Figure S3 in Supplementary Data) revealed that the *ermA* and *ermB* genes could not be detected among all case 47 isolates. However, *ermC* genes were detected in 18 isolates with the correct amplicons size, (142 bp) and (299 bp), respectively. A total of nineteen isolates were resistant to tetracycline (Table 10). The *tetK* gene was detected in 19 isolates, with the correct amplicon size of 360 bp. However, the *tetM* gene was not detected in any of the *S. aureus* isolates (N = 47). The penicillin resistance gene (*blaZ*) was detected in all the isolates of *S. aureus* (N = 47), with the expected amplicon size (173 bp). The oxacillin resistance gene (*mecA*) was detected in all the isolates of *S. aureus* isolates (N = 47), with the expected amplicon size (314 bp).

## 4. Discussion

*S. aureus* is a significant human pathogen both in hospitals and the community. This bacterium is an opportunistic pathogen responsible for a many self-limiting and even life-threatening diseases in humans. MRSA strains are common causes of emerging nosocomial infections and are considered to be a significant problem for public health. *S. aureus* is the leading cause of bloodstream infection in the majority of developed countries [20]. Virulence genes, such as enzymes, toxins, adhesin proteins, and cell surface proteins produced by *S. aureus*, play an important role in the pathogenicity of *S. aureus* strains. Virulence factors play different roles in various diseases [21]. Patients with SCD are known to have an increased risk of infections. SCD is a comprehensive term that refers to a group of autosomal genetic disorders associated with structurally abnormal hemoglobin [3]. The current study determined the incidence of some of the various virulence markers of *S. aureus* in the blood samples of SCD patients. The purpose of this study was to assess the prevalence of pathogenic *S. aureus* strains isolated from the blood of SCD patients who visited KSUMC. The susceptibility testing was performed with 26 different antibiotics. The PCR technique was used to detect the presence of the virulence genes clfA, fnbA, fnbB, sdrC, sdrD, spa, sea, seb, sec, sed, hla, hlg, pvl, tst, coa, efb, icaA, nuc, and agr. Furthermore, antibiotic resistance genes (plaZ, mecA, ermA, ermB, ermC, tetM, and tetK) were investigated.

In 2011, Jastaniah reported that SCD had spread throughout the Kingdom of Saudi Arabia, and the number of recorded cases varied by location, with the majority occurring in the eastern and southwestern regions. In some locations, the prevalence of this disease may approach 27% [22]. Retrospective research conducted by Zuair and his colleagues at KSUMC in 2023 revealed that, between 2016 and 2021, 160 adult patients with SCD visited the hospital for treatment [23]. Our current results are considerably different from this one, and the explanation for the considerable discrepancy in the number of cases is that Zuair and his colleagues’ study only targeted adults and employed a different approach to the one we applied. The findings of this work reported that all the isolates were ampicillin-resistant *S. aureus* strains. According to these results, this antibiotic is a completely excluded option for SCD patients infected with *S. aureus*. The majority of the isolates were also resistant to penicillin, which is almost consistent with the results of the ampicillin susceptibility test.

One of the most significant findings in this investigation was that the isolated *S. aureus* strains were all susceptible to the following antibiotics: fosfomycin, tigecycline; Synercid, mupirocin, linezolid; daptomycin, teicoplanin, vancomycin, and gentamicin. These antibiotics belong to several categories (aminoglycoside, glycylcycline, lipopeptide, and glycopeptide antibiotics) and act with different mechanisms (ligase inhibitors, protein synthesis inhibitors, and cell wall synthesis inhibitors), which makes them the best candidates for treatment, as well as for studying the synergistic action between them [24,25].

These results revealed that there was significant variation in the antibiotic susceptibility among the various *S aureus* strains. In contrast, in 2008, Campbell and his colleagues reported a high resistance of *S. aureus* strains to penicillin, tetracycline, and erythromycin that have been reported in Hatay, Turkey [16]. The variation in this resistance was observed clearly in some antibiotics for *S. aureus* in SCD patients. This result has been supported by studies performed in both the human and poultry sectors [3]. However, our results were in agreement with other studies that were carried out in Kuwait [26]. However, our results were slightly different from those reported in Accra (Ghana), which mentioned that there was variation in resistance observed clearly in some antibiotics for *S*, *aureus* in patients with SCD, penicillin (100%), cotrimoxazole (27.5%), tetracycline (25%), rifampicin (82.5%), erythromycin (30%), clindamycin (32.5%), gentamicin (7.5%), cefoxitin (27.5%), linezolid (30%), and fusidic acid (95%) [3]. Among the patients with SCD and control groups, respectively, there was significant variation in antibiotic susceptibility. This finding of antibiotic resistance was slightly different from that in other studies [27], including one from Tahran, which reported that the highest antibiotic resistance in *MRSA* was to penicillin, clindamycin, tobramycin, and tetracycline, respectively.

Bacterial pathogens often possess unique genes known as virulence factors, which allow them to cause damage or disease in their host and distinguish them from non-pathogenic organisms [28]. *S. aureus* is a highly virulent pathogen that is capable of infecting almost any part of the body. These factors can be divided into different groups, including surface-associated factors, toxins, enzymes, and hemolysins. Some *S. aureus* isolates secrete toxin superantigens, such as toxic shock syndrome toxin 1, exfoliative toxins, and enterotoxins. Superantigens can activate large numbers of T lymphocytes with a massive production of cytokines and chemokines. Hemolysins are cytotoxins that kill erythrocytes and/or phagocytes by forming pores in cell membranes and also provide the nutrients required for the growth and spreading of the pathogen throughout the body. Additionally, staphyloxanthin, a golden pigment produced by most *S. aureus* isolates, has been suggested to act as an important virulence factor and its inhibition has been shown to reduce the virulence of S. aureus [29]. Interestingly, strong correlations were detected between the presence of the *finbB*, *sdrC*, *sdrD*, *icaA*, *coa*, *nuc*, *hlg*, *hla*, *finbA*, *clfA*, and *efb* genes and *S. aureus* among the SCA blood samples, thus indicating a high level of genetic diversity among the study population. These results indicate that, in the future, we need to monitor the genes that are absent or present at low levels in these patients. The most prevalent virulence genes in the studied population were *finbB*, *sdrC*, *sdrD*, *icaA*, *coa*, *nuc*, *hlg*, *hla*, *finbA*, *clfA*, and *efb*, followed by *pvl*, *agr*, *spa*, *seb*, *sea*, *sec*, and *tst*, respectively. This finding is consistent with the results in [20], which found that all the isolates from their study population had a combination of the sdrC, icaA, hla, clf, and sdrD genes. In this study, we observed the presence of the sea gene in 25.5% of the *S. aureus* strains. However, our results are in contrast to another study carried out by Yu and his colleagues (2012) in four hospitals in the Zhejiang province, eastern China, which found that the sea gene was present in 68.5% of the *S. aureus* strains from their study population. The difference in these results may be due to differences in the study populations and geographical areas

The *hlg* (γ-haemolysin) gene was present in 100% of the isolates in this study. This finding result was in agreement with Elboshra and his colleagues (2020) [30], who found that the presence of the *hlg* gene is strongly associated with virulence in MRSA, as the γ toxins produced by the gene can help the bacteria to evade the immune system and persist in the host, leading to more severe infections and treatment challenges.

The *seb* gene is known to contribute to the pathogenesis of a range of diseases caused by *S. aureus*, including pneumonia, toxic shock syndrome, and sepsis, and has been implicated in the exacerbation of community-associated (CA) MRSA infections. Its ability to activate immune cells and cause a cytokine storm can lead to tissue damage and organ failure, making *seb* a potential target for immunomodulatory therapies [31]. In our study, was observed the presence of the *seb* gene in approximately 27.7% of the *S. aureus* strains, which is in complete agreement with the study conducted by [32] in Iran. Moreover, a significant association was observed between the case and control for the presence of the *seb* gene carriage (*p* < 0.0001). In addition, all the isolates of MRSA strains showed diverse combinations of the *ica*, *hla*, *hlg*, and *coa* genes, thus indicating a significant level of genetic diversity within the study population.

*S. aureus* toxins, including α-toxin, β-toxin, and *pvl*, are critical in causing pneumonia and lung injury. These pore-forming toxins exacerbate a host’s inflammatory response, inducing the expression of proinflammatory cytokines and releasing additional inflammatory mediators. Their direct and indirect mechanisms cause lung damage, but their significance in *S. aureus*-induced pneumonia and lung injury remains unclear [33]. In our study, was observed the presence of the *pvl* gene in approximately 53.2% of the S. *aureus* isolates. These results are consistent with the study conducted in Riyadh, Saudi Arabia, by [34], which reported a remarkably high prevalence of the *pvl* gene (54.21%) in MRSA.

The *mecA* gene codes for a protein called PBP2a, which is responsible for conferring resistance to methicillin and other drugs in the β-lactam group. PBP2a has a low affinity for β-lactam antibiotics, allowing for bacteria carrying the *mecA* gene to survive in their presence. PBP2a can also confer cross-resistance to other drugs in the β-lactam group, making it difficult to treat infections caused by bacteria with the *mecA* gene [35]. It is a genetic element that harbors the methicillin resistance (*mecA*) gene and is more easily transferred to other strains of *S. aureus* [36]. Our study found that approximately 93.3% of the *mecA*-positive isolates were resistant to oxacillin, a finding that is consistent with a previous study by [6], where 86.8% of *mecA*-positive isolates obtained from processed food samples in Riyadh, Saudi Arabia, were found to be resistant to oxacillin. Moreover, a significant association was observed between the case and control for the presence of the *mecA* gene carriage (*p* < 0.0001).

The main limitation of our study is that we investigated just 19 of the *S. aureus* virulence factor genes and 7 of the *S. aureus* antibiotic resistance genes. These were the ones with which our microbiologist had experience and that we could assess in our laboratory. Thus, we did not test some virulence factors that are important in other studies on the SCA disease. Another limitation is the small size of our sample. The strengths of this investigation include the fact that it is a prospective study, all the samples for culture were taken from the blood, and every patient classified as having SCA had a definitive pathological diagnosis.

## 5. Conclusions

In conclusion, to the best of our knowledge, this is the first study in Saudi Arabia to study the distribution of virulence genes and antibiotic resistance genes among patients with SCA. Our study indicated the presence of several virulence factors and resistance to antibiotics in clinical *S. aureus* and MRSA isolates among SCD patients. The finbB, *sdrC*, *sdrD*, *icaA*, *coa*, *nuc*, *hlg*, *hla*, *finbA*, *clfA*, *efb*, *icaA*, *pvl*, *agr*, and *spa* genes were predominant in the isolates. The relationship between the virulence genes and resistance to antibiotics showed that the highest resistance was observed in the isolates with the *finbB*, *sdrC*, *sdrD*, *icaA*, *coa*, *nuc*, *hlg*, *hla*, *finbA*, *clfA*, *efb*, and *icaA* genes. There were significant relationships between *S. aureus* and MRSA and the presence of the *finbB*, *sdrC*, *sdrD*, *icaA*, *coa*, *nuc*, *hlg*, *hla*, *finbA*, *clfA*, *efb*, and *icaA* genes (*p<* 0.01). Our results showed the presence of the *mecA* and *plaZ* genes among all the isolates. However, the *ermA*, *ermB*, and *tetM* genes could not be detected among all case 47 isolates. Moreover, a significant association was observed between the case and the control and the presence of the *mecA* gene (*p* < 0.0001). Our findings indicated a high incidence of *finbB*, *sdrC*, *sdrD*, *icaA*, *coa*, *nuc*, *hlg*, *hla*, *finbA*, *clfA*, *efb*, and *icaA*—positive and MRSA strains with higher rates of antibiotic resistance.

The obtained results of this study can be further improved by taking into consideration the sequencing of the resistant *mecA* gene to assess the genetic variation and molecular typing for clonality, which are fundamental in determining and influencing the resistance profile of isolates globally. In addition, our research conducted on the virulence factors of an antibiotic-resistant *S. aureus* was restricted to the KSUMC hospital. Further investigations are needed to examine other strains from patients with SCA, and advanced methods should be utilized to detect the virulence genes in such strains. These issues should be the subject of future research.

## Figures and Tables

**Table 1:** Virulence genes primers used in this study, target gene, and sequence.

Gene Target	5′—Oligo Seq—3′	Product Size (bp)	Annealing Temp. (°C)	Ref.
*fnbA*-F	CACAACCAGCAAATATAG	1279	48	[17]
*fnbA*-R	CTGTGTGGTAATCAATGTC			
*fnbB*-F	CAGAAGTACCAAGCGAGCCGGAAA	258	65	[16]
*fnbB*-R	CGAACAACATGCCGTTGTTTGTTGA			
*clfA*-F	ATTGGCGTGGCTTCAGTGCTTG	357	48	[16]
*clfA*-R	GCTTGATTGAGTTGTTGCCGGTGT			
*sdrC*-F	CGCATGGCAGTGAATACTGTTGCAGC	731	50	[17]
*sdrC*-R	GAAGTATCAGGGGTGAAACTATCCACAAATTG			
*sdrD*-F	CCACTGGAAATAAAGTTGAAGTTTCAACTGCC	467	61	[17]
*sdrD*-R	CCTGATTTAACTTTGTCATCAACTGTAATTTGTG			
*spa*-F	TCGAAATAGCGTGATTTTGC	polymorphism	61	[17]
*spa*-R	GCACTGAGCAACAAAAGATG			
*pvl*-F	TGCCAGACAATGAATTACCCCCATT	894	60	[17]
*pvl*-R	TCTGCCATATGGTCCCCAACCA			
*sea*-F	TTGCAGGGAACAGCTTTAGGCAATC	252	60	[17]
*sea*-R	TGGTGTACCACCCGCACATTGA			
*seb*-F	GACATGATGCCTGCACCAGGAGA	355	64	[17]
*seb*-R	AACAAATCGTTAAAAACGGCGACACAG			
*sec*-F	CCCTACGCCAGATGAGTTGCACA	602	62	[17]
*sec*-R	CGCCTGGTGCAGGCATCATATC			
*sed*-F	GAAAGTGAGCAAGTTGGATAGATTGCGGCTAG	362	65	[17]
*sed*-R	CCGCGCTGTATTTTTCCTCCGAGAG			
*agr* I-F	ATCGCAGCTTATAGTACTTGT	814	55	[16]
*agr* I-R	CTTGATTACGTTTATATTTCATC			
*efb*-F	CGGTCCAAGAGAAAAGAAACCAGTGAG	276	63	[17]
*efb*-R	TGTGCTTTTCTGTGTGCACTGACAGTATG			
*icaA*-F	TCAGACACTTGCTGGCGCAGTC	936	55	[17]
*icaA* -R	TCACGATTCTCTCCCTCTCTGCCATT			
*tst*-F	AGCCCTGCTTTTACAAAAGGGGAAAA	306	64	[17]
*tst*-R	CCAATAACCACCCGTTTTATCGCTTG			
*hlg*-F	TTGGCTGGGGAGTTGAAGCACA	306	64	[17]
*hlg*-R	CGCCTGCCCAGTAGAAGCCATT			
*hla*-F	CGAAAGGTACCATTGCTGGT	744	53	[18]
*hla*-R	CCAATCGATTTTATATCTTTC			
*coa* -F	ATAGAGATGCTGGTACAGG	polymorphism	58	[17]
*coa*-R	GCTTCCGATTGTTCGATGC			
*nuc*-F	GCGATTGATGGTGATACGGTT	279	60	[17]
*nuc*-R	AGCCAAGCCTTGACGAACTAAAGC			

**Table 2:** Primers sequence specific to different antimicrobials resistant determinants in *S. aureus*.

Gene Target	5′—Oligo Seq—3′	Product Size (bp)	Annealing Temp. (°C)	Ref.
*blaZ*-F	ACTTCAACACCTGCTGCTTTC	173	54	[14]
*blaZ*-R	TGACCACTTTTATCAGCAACC			
*mecA*-F	CCTAGTAAAGCTCCGGAA	314	55	[14]
*mecA*-R	CTAGTCCATTCGGTCCA			
*tetK*-F	GTAGCGACAATAGGTAATAGT	360	54	[14]
*tetK*-R	GTAGTGACAATAAAC CTCCTA			
*tetM*-F	AGTGGAGCGATTACAGAA	158	54	[14]
*tetM*-R	CATATGTCCTGG CGTGTCTA			
*ermA*-F	AAGCGGTAAACCCCTCTG A	190	58	[14]
*ermA*-R	TTC GCAAATCCCTTCTCAAC			
*ermB*-F	CTATCTGATTGTTGAAGAAGGATT	142	54	[14]
*ermB*-R	GTTTACTCTTGGTTTAGGATGAAA			
*ermC*-F	AAT CGTCAA TTCCTG CAT GT	299	54	[14]
*ermC*-R	TAATCG TGGAATACGGGTTTG			
16S-F	AGA GTT TGA TCC TGG CTC AG	1500	52	[19]
16S-R	AAG GAG GTG ATC CAG CCG CA			

**Table 3:** Antibiotic susceptibility pattern of *S. aureus* strains (case) isolated from patients with SCD (HbSS) (N = 47) compared with *S. aureus* strains (control) isolated from individuals without SCD disease (N = 16).

Antibiotic	Group	Sensitive (N, %)	Resistance (N, %)	*p* Values *
Amox	Case ^1^	13 (27.7)	34 (72.3)	0.001
	Control ^2^	12 (75)	4 (25)	
Amp	Case	0 (0.0)	47 (100)	0.000
	Control	0 (0.0)	16 (100)	
Azith	Case	38 (80.9)	9 (19.1)	0.545
	Control	14 (87.5)	2 (12.5)	
Cefaz	Case	15 (31.9)	32 (68.1)	0.031
	Control	10 (62.5)	6 (37.5)	
Cipro	Case	39 (83)	8 (17)	0.669
	Control	14 (87.5)	2 (12.5)	
Clinda	Case	42 (89.9)	5 (4.3)	0.838
	Control	14 (87.5)	2 (12.5)	
Dapto	Case	47 (100)	0 (0)	0.000
	Control	16 (100)	0 (0)	
Eryth	Case	30 (63.8)	17 (36.2)	0.196
	Control	13 (81.3)	3 (18.8)	
Fosfo	Case	47 (100)	0 (0)	0.000
	Control	16 (100)	0 (0)	
Fusa	Case	39 (83)	4 (8.9)	0.869
	Control	13 (81.3)	2 (12.5)	
Gent	Case	47 (100)	0 (0)	0.084
	Control	15 (93.8)	1 (6.3)	
Imi	Case	15 (31.9)	32 (68.1)	0.003
	Control	12 (75)	4 (25)	
Levo	Case	42 (89.4)	5 (10.6)	0.4
	Control	13 (81.3)	3 (18.7)	
Lzd	Case	47 (100)	0	0.000
	Control	16 (100)	0	
Moxi	Case	40 (85.1)	7 (14.9)	0.358
	Control	12 (75)	4 (25)	
Mup	Case	47 (1000)	0 (0)	0.000
	Control	16 (100)	0 (0)	
Ox	Case	16 (34)	31 (66)	0.004
	Control	12 (75)	4 (25)	
Pen	Case	1 (2.1)	46 (97.9)	0.556
	Control	0 (0)	16 (100)	
Rif	Case	24 (51.1)	2 (4.3)	0.010
	Control	15 (93.8)	0 (0)	
SXT	Case	45 (95.7)	2 (4.3)	0.746
	Control	15 (93.8)	1 (6.3)	
Synercid	Case	47 (100)	0 (0)	0.000
	Control	16 (100)	0 (0)	
Teico	Case	47 (100)	0 (0)	0.000
	Control	16 (100)	0 (0)	
Tetra	Case	36 (76.6)	11 (23.4)	0.35
	Control	14 (87.5)	2 (12.5)	
Tgc	Case	47 (100)	0 (0)	0.000
	Control	16 (100)	0 (0)	
Tobra	Case	42 (89.4)	5 (10.6)	0.606
	Control	15 (93.8)	1 (6.3)	
Vanc	Case	44 (93.6)	3 (6.4)	0.434
	Control	14 (87.5)	2 (12.5)	

* The resistance rates of antibiotics among *S. aureus* compared with the sensitivity of those antibiotics among patients and control. ^1^ Case: 47 strains of *S. aureus* were isolated from patients with SCD (HbSS). ^2^ Control: 16 strains of *S. aureus* without the SCD disease.

**Table 4:** Correlation of antibiotic susceptibility of *S. aureus* isolates (N = 47) between *MRSA* (N = 30) and *S. aureus* (N = 17).

Antibiotic	Type of Bacteria	Sensitive (N, %)	Resistance (N, %)	*p* Values *
Amox	MRSA ^1^	0 (0)	30 (100)	0.000
	S.A ^2^	13 (76.5)	4 (23.5)	
Amp	MRSA	0 (0)	30 (100)	0.000
	S.A	0 (0)	17 (100)	
Azith	MRSA	23 (76.7)	7 (23.3)	0.333
	S.A	15 (88.2)	2 (11.8)	
Cefaz	MRSA	0 (0)	30 (100)	0.000
	S.A	15 (88.2)	2 (11.8)	
Cipro	MRSA	24 (80)	6 (20)	0.470
	S.A	15 (88.2)	2 (11.8)	
Clinda	MRSA	25 (83.3)	5 (16.7)	0.075
	S.A	17 (100)	0 (0)	
Dapto	MRSA	30 (100)	0 (0)	0.072
	S.A	17 (100)	0 (0)	
Eryth	MRSA	21 (70)	9 (30)	0.242
	S.A.	9 (52.9)	8 (47.1)	
Fosfo	MRSA	30 (100)	0 (0)	0.447
	S.A	17 (100)	0 (0)	
Fusa	MRSA	27 (90)	0 (0)	0.019
	S.A	11 (64.7)	4 (23.5)	
Gent	MRSA	30 (100)	30 (0)	0.000
	S.A	17 (100)	17 (0)	
Imi	MRSA	0 (0)	30 (100)	0.000
	S.A	15 (88.2)	2 (11.8)	
Levo	MRSA	25 (83.3)	5 (16.7)	0.075
	S.A	17 (100)	0 (0)	
Lzd	MRSA	30 (100)	0 (0)	0.012
	S.A	17 (100)	0 (0)	
Moxi	MRSA	24 (80)	6 (20)	0.191
	S.A	16 (94.1)	1 (5.9)	
Mup	MRSA	30 (100)	30 (0)	0.277
	S.A	17 (100)	17 (0)	
Ox	MRSA	0 (0)	30 (100)	0.000
	S.A	16 (94.1)	1 (5.9)	
Pen	MRSA	0 (0)	30 (100)	0.179
	S.A	1 (5.9)	16 (94.1)	
Rif	MRSA	19 (63.3)	1 (3.3)	0.082
	S.A	5 (29.4)	1 (5.9)	
Sxt	MRSA	30 (100)	0 (0)	0.000
	S.A	15 (88.2)	2 (11.8)	
Synercid	MRSA	30 (100)	0 (0)	0.000
	S.A	17 (100)	0 (0)	
Teico	MRSA	30 (100)	0 (0)	0.000
	S.A	17 (100)	0 (0)	
Tetra	MRSA	20 (66.7)	10 (33.3)	0.033
	S.A	16 (94.1)	1 (5.9)	
Tgc	MRSA	30 (100	0 (0)	0.000
	S.A	17 (100)	0 (0)	
Tobra	MRSA	29 (96.7)	1 (3.3)	0.031
	S.A	13 (76.5)	4 (23.5)	
Vanc	MRSA	29 (96.7)	1 (3.3)	0.256
	S.A	15 (88.2)	2 (11.8)	

^1^ Methicillin resistant *S. aureus*. ^2^ Non Methicillin resistant *S. aureus*. * The correlation rates antibiotic susceptibility between cases and control.

**Table 5:** Correlation of positive virulence genes of *S. aureus* isolates (N = 47) in MRSA (N = 30) and S.A (N = 17) and sex, male (N = 30) and female (N = 17).

Variables	Positive Gene, Positive %	Type of Bacteria				*p* Values *
Gene	Number 47	MRSA		S. A		
		Male	Female	Male	Female	
*finbA*	46 (97.9)	20	9	9	8	0.277
*sdrC*	47 (100)	21	9	9	8	0.242
*sea*	12 (25.5)	8	4	0	0	0.213
*tst*	3 (4.3)	2	0	0	1	0.083
*clfA*	46 (97.9)	21	9	8	8	0.181
*coa*	47 (100)	21	9	9	8	0.242
*spa*	20 (42.6)	9	4	3	4	0.251
*finbB*	47 (100)	21	9	9	8	0.242
*agr*	22 (46.8)	10	6	2	3	0.375
*nuc*	47 (100)	21	9	9	8	0.242
*efb*	46 97.9)	20	9	9	8	0.277
*pvl*	25 (53.2)	13	3	8	2	0.937
*sdrD*	47 (100)	21	9	9	8	0.242
*hlg*	47 (100)	21	9	9	8	0.242
*hla*	47 (100)	21	9	9	8	0.242
*seb*	13 (27.7)	8	4	0	1	0.188
*sec*	6 (12.8)	0	3	1	2	0.273
*sed*	2 (4.3)	1	0	1	0	1
*icaA*	47 (100	21	9	9	8	0.242

*p* values *: Correlation between positive gene and type of bacteria (MRSA and SA). *finbA* and *finbB*: Fibronectin-binding proteins. *SdrC* and *sdrD*: Serine Aspartic Repeat Protein. *ClfA*: Clumping factor proteins. *sea*, *seb*, *sec*, and *sed*: Staphylococcal enterotoxins. *hlg*. *hla*, *pvl*, and tst: exotoxin genes. *nuc*: Nucleases. *coa*, *efb*, *icaA*, and *agr*: other virulence genes in *S. aureus.*

**Table 6:** Correlation of *S. aureus* virulence genes between patients (N = 47) isolates and control (N = 16) isolates.

Gene	Group	Positive N, Positive%	* *p*-Value	Negative N, Negative%	** *p*-Value
*finbA*	Case	46 (97.9)	0.05	1 (2.1)	0.019
	Control	13 (81.3)		3 (18.8)	
*sdrC*	Case	47 (100)	1	0 (0)	0.000
	Control	16 (100)		0 (0)	
*sea*	Case	12 (25.5)	0.2	35 (74.5)	0.279
	Control	2 (12.5)		14 (87.5)	
*tst*	Case	2 (4.3)	0.3	45 (95.7)	0.243
	Control	2 (12.5)		14 (87.5)	
*clfA*	Case	46 (97.9)	0.3	1 (2.1)	0.556
	Control	16 (100)		0 (0)	
*coa*	Case	47 (100)	0.05	0 (0)	0.002
	Control	13 (81.3)		3 (18.8)	
*spa*	Case	20 (42.6)	0.9	27 (57.4)	0.933
	Control	7 (43.8)		9 (56.3)	
*finbB*	Case	47 (100)	1	0 (0)	0.000
	Control	16 (100)		0 (0)	
*agr*	Case	22 (46.8)	0.03	25 (53.2)	0.051
	Control	12 (75)		4 (25)	
*nuc*	Case	47 (100)	1	0 (0)	0.000
	Control	16 (100)		0 (0)	
*efb*	Case	46 (97.9)	0.2	1 (2.1)	0.092
	Control	14 (87.5)		2 912.5)	
*pvl*	Case	25 (54.3)	0.8	21 (45.7)	0.895
	Control	9 (56.3)		7 (43.8)	
*sdrD*	Case	47 (100)	0.3	0 (0)	0.084
	Control	15 (93.8)		1 (6.3)	
*hlg*	Case	47 (100)	1	0 (0)	0.000
	Control	16 (100)		0 (0)	
*hla*	Case	47 (100)	1	0 (0)	0.000
	Control	16 (100)		0 (0)	
*seb*	Case	13 (27.7)	0.00	34 (72.3)	0.018
	Control	0 (0)		16 (100)	
*sec*	Case	6 (12.8)	0.4	41 (87.2)	0.474
	Control	1 (6.3)		15 (93.8)	
*sed*	Case	2 (4.3)	0.1	45 (95.7)	0.402
	Control	0 (0)		16 (100)	
*icaA*	Case	47 (100)	0.1	0 (0)	0.014
	Control	14 (87.5)		2 (12.5)	

* *p*-value: correlation between case and control in a positive gene. ** *p*-value: correlation between case and control in positive gene compared with negative gene.

**Table 7:** Correlation of *S. aureus* virulence genes between MRSA (N = 30) isolates and non-MRSA (S.A) (N = 10) isolates.

Gene	Type of Bacteria	Positive N, Positive%	* *p*-Value	Negative N, Negative%	** *p*-Value
*finbA*	MRSA	29 (96.7)	0.30	1(3.3%)	0.447
	S.A	17 (100)		0 (0.0%)	
*sdrC*	MRSA	30 (100)	1	0 (0)	0.000
	S.A	17 (100)		0 (0)	
*sea*	MRSA	12 (40)	0.0000	18 (60)	0.003
	S.A	0 (0)		17 (100)	
*tst*	MRSA	2 (6.7)	0.14	28 (93.3)	0.277
	S.A	0 (0)		17 (100)	
*clfA*	MRSA	30 (100)	0.3	0 (0)	0.179
	S.A	16 (94.1)		1 (5.9)	
*coa*	MRSA	30 (100)	1	0 (0)	0.000
	S.A	17 (100)		0 (0)	
*spa*	MRSA	13 (43.3)	0.88	17 (56.7)	0.886
	S.A	7 (41.2)		10 (58.8)	
*finbB*	MRSA	30 (100)	1	0 (0)	0.000
	S.A	17 (100)		0 (0)	
*agr*	MRSA	17 (56.7)	0.056	13 (43.3)	0.072
	S.A	5 (29.4)		12 (70.6)	
*nuc*	MRSA	30 (100)	1	0 (0)	0.000
	S.A.	17 (100)		0 (0)	
*efb*	MRSA	29 (96.7)	0.3	1 (3.3)	0.447
	S.A	17 (1000		0 (0)	
*pvl*	MRSA	15 (51.7)	0.5	14 (48.3)	0.64
	S.A	10 (58.8)		7 (41.2)	
*sdrD*	MRSA	30 (100)	1	0 (0)	0.000
	S.A	17 (100)		0 (0)	
*hlg*	MRSA	30 (100)	1	0 (0)	0.000
	S.A	17 (100)		0 (0)	
*hla*	MRSA	30 (100)	1	0 (0)	0.000
	S.A	17 (100)		0 (0)	
*seb*	MRSA	12 (40)	0.001	18 (60)	0.012
	S.A	1 (5.9)		16 (94.1)	
*sec*	MRSA	3 (10)	0.4	27 (90)	0.450
	S.A	3 (17.6)		14 (82.4)	
*sed*	MRSA	2 (6.7)	0.1	28 (93.3)	0.277
	SA	0 (0)		0 (0)	
*icaA*	MRSA	30 (1000	1	0 (0)	0.000
	SA	17 (100)		0 (0)	

* *p*-value: correlation between MRSA and *S. aureus* in a positive gene. ** *p*-value: correlation between MRSA and *S. aureus* in positive gene compared with negative gene.

**Table 8:** Comparison of the antibiotic-resistant cases in the presence of a positive gene with antibiotic-resistant cases in a negative gene.

Gene	Antibiotic	B	S.E.	Wald	df	Sig. *	Exp(B) (R)	Model ** *p*-Value	Nagelkerke R Square
*seb*	Ox	2.25	1.1	4.2	1	0.040	9.5	0.010	18.8%
	Amox	1.9	1.1	2.9	1	0.050	6.6	0.040	12.4%
*agr*	Cefaz	1.6	0.7	4.7	1	0.030	4.9	0.020	14.6%
*sea*	Ox	20	10,048	0.00	1	0.999	102,099	0.001	33.2%
	Amox	20	11,147	0.00	1	0.999	881,168	0.002	26.3%
	Imi	20	10,377	0.00	1	0.999	969,284	0.001	30%
	Cefaz	20	10,742	0.00	1	0.998	881,168	0.001	28%

* Correlation between antibiotic resistance and the presence of genes in *S. aureus* isolates: a comparison of resistance in gene-positive vs. gene-negative strains. **: Correlation between antibiotic resistance and positive gene.

**Table 9:** Prevalence of virulence genes (%) according to antibiotic resistance in 47 *S. aureus* isolates.

Gene	Amox	Amp	Azith	Cefaz	Cipro	Clinda	Eryth	FusA	Imi	Levo	Moxi	Ox	Pen	Rif	SXT	Tetra	Tobra
*fnbA*	71.7 *	100 *	19.6	69.6 *	17.4	10.9	37	8.7 **	67.4 *	10.9 **	15.2	65.2 *	97.8 *	4.3 **	4.3 **	6.4 **	10.6
*sdrC*	72.3 *	100 *	19.1	70.2 *	17	10.6	36.2	8.5 **	68.1 *	10.6 **	14.9	66 *	97.9 *	4.3 **	4.3 **	23.4	10.6
*sea*	100 *	100 *	33.3	100 *	8.3 **	16.7	58.3	0.0 **	100 *	8.3 **	16.7	100 *	100 *	8.3 **	0.0 **	33.3	8.3 **
*tst*	100 *	100 **	50	100 *	0.0 **	50	50**	0.0 **	100 *	0.0 **	0.0 **	100 *	100 **	0.0 **	0.0 **	50 **	0.0 **
*clfA*	73.9 *	100 *	19.6	69.6 *	17.4	10.9	37	8.7 **	69.6 *	10.9	15.2	67.4 *	97.8 *	4.3 **	4.3 **	23.9	10.9
*coa*	72.3 *	100 *	19.1	70.2 *	17	10.6	36.2	8.5 **	68.1 *	10.6	14.9	66 *	97.9 *	4.3 **	4.3 **	23.9	10.6
*spa*	65 *	100 *	15	70 *	5 **	15	45	15	70 *	10	15	65 *	100 *	0.0 **	5 **	35	15
*fnbB*	72.3 *	100 *	19.1	70.2 *	17	10.6	36.2	8.5 **	68.1 *	10.6	14.9	66 *	97.9 *	4.3 **	4.3 **	23.4	10.6
*agr*	77.3 *	100 *	22.7	86.4 *	18.2	9.1	40.9	4.5 **	77.3 *	13.6	18.2	77.3 *	100 *	4.5 **	0.0 **	13.6	9.1
*nuc*	72.3	100 *	19.1	70.2 *	17	10.6	36.2	8.5 **	68.1 *	10.6	14.9	66 *	97.9 *	4.3 **	4.3 **	23.4	10.6
*efb*	71.7 *	100 *	19.6	69.6 *	17.4	10.9	34.8	8.7 **	67.4 *	10.9	15.2	65.2 *	97.8 *	4.3 **	4.3 **	21.7	10.9
*pvl*	72 *	100 *	20	72 *	16	4 **	24	4 **	64 *	4 **	12	64 *	96 *	8 **	0.0 **	16	8
*sdrD*	72.3 *	100 *	19.1	70.2 *	17	10.6	36.2	8.5 **	68.1 *	10.6	14.9	66 *	97.9 *	4.3 **	4.3 **	23.4	10.6
*hlg*	72.3 *	100 *	19.1	70.2 *	17	10.6	36.2	8.5 **	68.1 *	10.6	14.9	66 *	97.9 *	4.3 **	4.3 **	23.4	10.6
*hla*	72.3 *	100 *	19.1	70.2 *	17	10.6	36.2	8.5 **	68.1 *	10.6	14.9	66 *	97.9 *	4.3 **	4.3 **	23.4	10.6
*seb*	92.3 *	100 *	15.4	92.3 *	23.1	15.4	30.8	0.0 **	92.3 *	30.8	30.8	92.3 *	100 *	7.7 **	0.0 **	30.8	0.0 **
*sec*	50	100 *	0.0 **	50	33.3	33.3	33.3	16.7	50	33.3	33.3	66.7 *	100 *	0.0 **	0.0 **	16.7	16.7
*icA*	72.3	100 *	19.1	70.2 *	17	10.6	36.2	8.5 **	68.1 *	10.6	14.9	66 *	97.9 *	4.3 **	4.3 **	23.4	10.6

*: High rates of antibiotic resistance were observed in S. aureus isolates, regardless of the presence of positive or negative virulence genes. ** Low rates of antibiotic resistance (sensitivity) in the presence of positive or negative virulence genes *S. aureus* isolates.

**Table 10:** Correlation of *S. aureus* antibiotic resistance genes between patients (N = 47) isolates and control (N = 16) isolates.

Gene	Group	Positive N, Positive%	Negative N, Negative%	*p* Values *
*blaZ*	Case	47 (100)	0 (0)	1
	Control	16 (100)	0 (0)	
*mecA*	Case	47 (100)	0 (0)	0.00
	Control	9 (56.3)	7 (43.7)	
*tetK*	Case	19 (40.4)	28 (59.6)	0.26
	Control	4 (25)	12 (75)	
*tetM*	Case	0 (0)	47 (100)	1
	Control	0 (0)	16 (100)	
*ermA*	Case	0 (0)	47 (100)	0.01
	Control	2 (12.5)	14 (87.5)	
*ermB*	Case	0 (0)	47 (100)	1
	Control	0 (0)	16 (100)	
*ermC*	Case	15 (31.9)	32 (68.1)	0.31
	Control	3 (18.8)	13 (81.2)	

* Correlation between *S. aureus* antibiotic resistance genes in case and control groups.

**Table 11:** Correlation of *S. aureus* antibiotic resistance genes between MRSA (N = 17) isolates and *S. aureus* (N = 10) isolates.

Gene	Type of Bacteria	Positive N, Positive%	Negative N, Negative%	*p* Values *
*blaZ*	MRSA	30 (100)	0 (0)	1
	SA	17 (100)	0 (0)	
*mecA*	MRSA	30(100)	0 (0)	1
	SA	17 (10)	0 (0)	
*tetK*	MRSA	17 (56.7)	13 (43.3)	0.003
	SA	2 (11.5)	15 (88.2)	
*tetM*	MRSA	0 (0)	30 (100)	1
	SA	0 (0)	17 (100)	
*ermA*	MRSA	0 (0)	30 (100)	1
	SA	0 (0)	17 (87.5)	
*ermB*	MRSA	0 (0)	30 (100)	1
	SA	0 (0)	17 (100)	
*rmC*	MRSA	8 (26.7)	22 (73.3)	0.31
	SA	7 (41.2)	10 (58.8)	

* Correlation between *S. aureus* antibiotic resistance genes in MRSA and SA bacteria.

## Data Availability

The data used in this study is available upon request from the corresponding author’s email.

## Supplementary Materials

The following supporting information can be downloaded at: https://www.mdpi.com/2079-6382/12/7/1221/s1, Figure S1: Agarose gel electrophoresis of amplified 16S rRNA gene of *S. aureus* isolates. Lane ladder: 250 bp, other lanes: PCR products of isolates for: (a) 20 samples; (b) 17 samples; (c) 10 samples, Figure S2: Agarose gel electrophoresis of the amplified adhesion-associated genes (*clfA*, *fnbA*, *fnbB*, *sdrC*, *sdrD*, and *spa*), enterotoxin A, B and C genes (sea, seb, sec) and other exotoxin genes (*hla*, *hlg*, *pvl* and *tst*), and others (*coa*, *efb*, *icaA*, *nuc* and *agr*) virulence genes. 25 µL of the PCR product was separated in 1% agarose containing ethidium bromide solution (1 µg/mL), and visualized using Gel documentation system. Lane ladder: 100 bp; Other lanes: *S. aureus* strain for different virulence genes, Figure S3: Agarose gel electrophoresis of the amplified *tetK*, *plaZ*, *mecA*, *ermA* and *ermC* antibiotics resistance genes. 25 µL of the PCR product was separated in 1% agarose containing ethidium bromide solution (1 µg/mL), and visualized using Gel documentation system. M: 100 bp ladder; other lanes: *S. aureus* strains.
