# Supplementary material for: The Prevalence of the Virulence Genes of Staphylococcus aureus in Sickle Cell Disease Patients at KSUMC, Riyadh, Saudi Arabia
Source: Antibiotics (Basel). 2023 Jul 22;12(7):1221. doi: 10.3390/antibiotics12071221 (PMC10416153; doi:10.3390/antibiotics12071221)
Supplement: Supplementary file 1 [file antibiotics-12-01221-s001.zip › antibiotics-2489001-supplementary.pdf]

Supplementary data

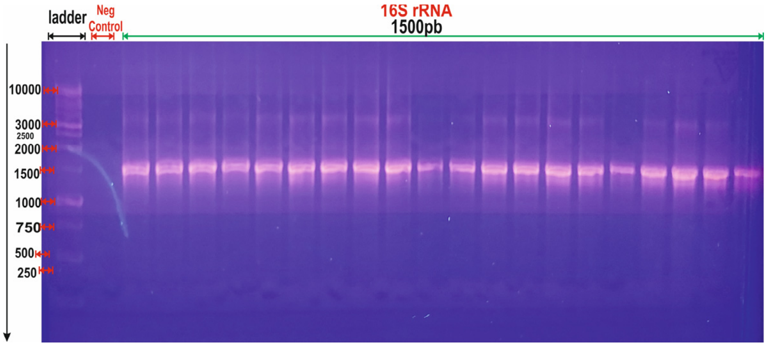

(**a**)

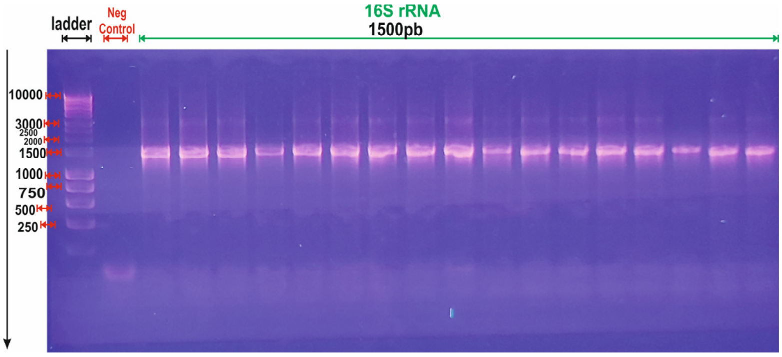

(**b**)

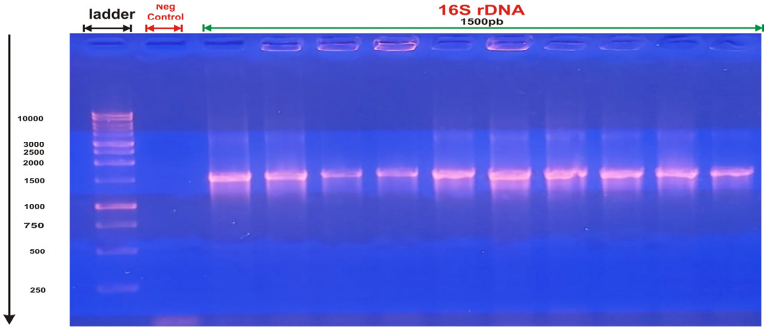

(**c**)

Figure S1. Agarose gel electrophoresis of amplified 16S rRNA gene of *S. aureus* isolates. Lane ladder: 250 bp, other lanes: PCR products of isolates for: (**a**) 20 samples; (**b**) 17 samples; (**c**) 10 samples.

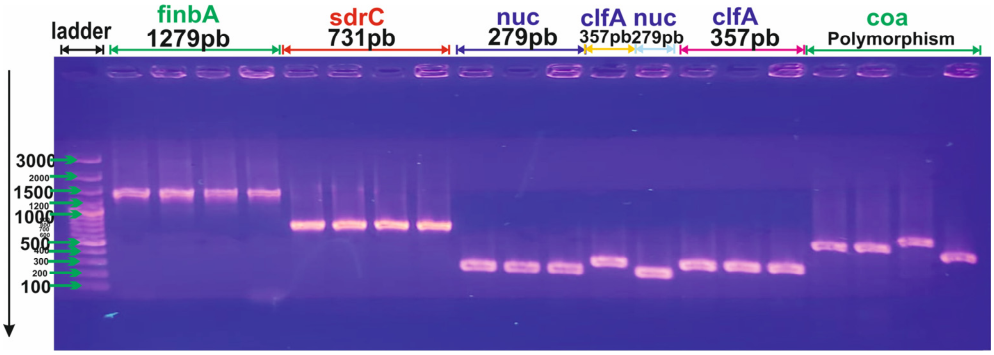

(**a**)

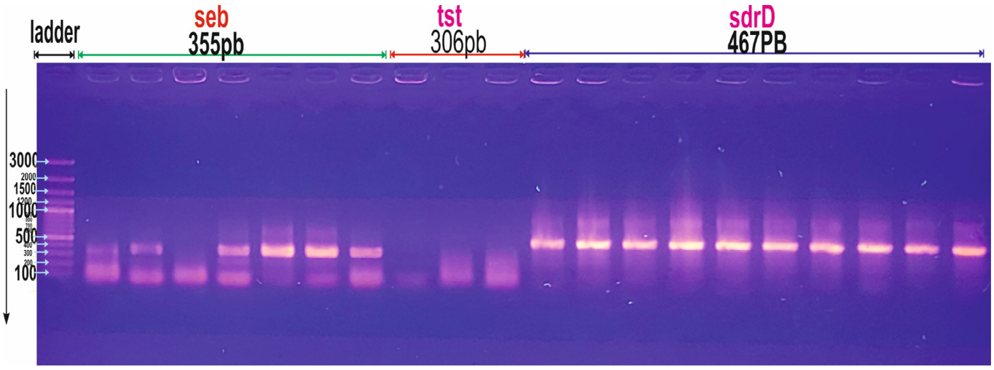

(**b**)

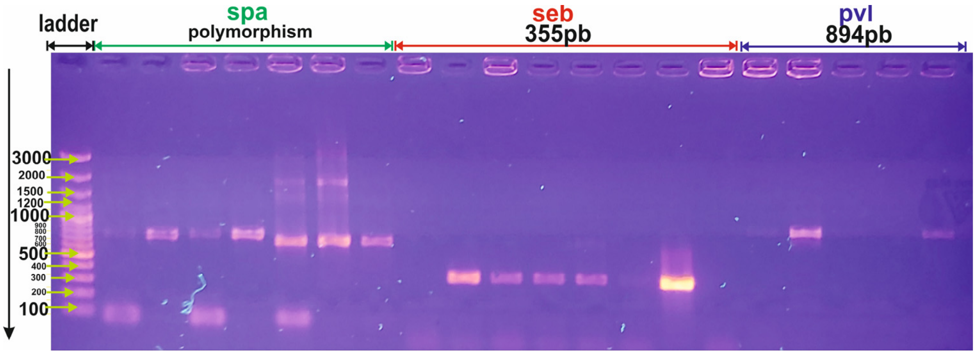

(**c**)

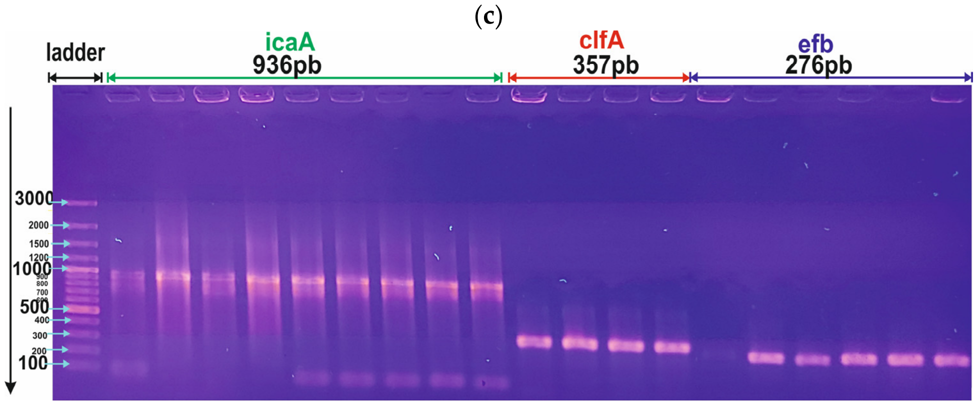

(d)

Figure S2. Cont.

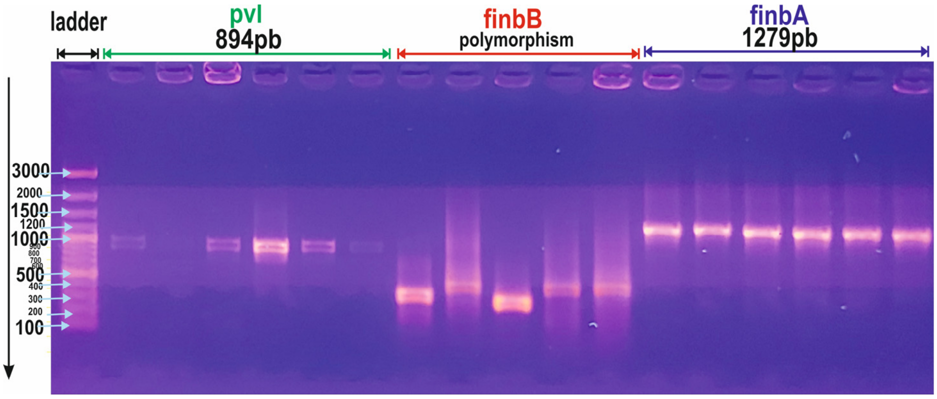

(**e**)

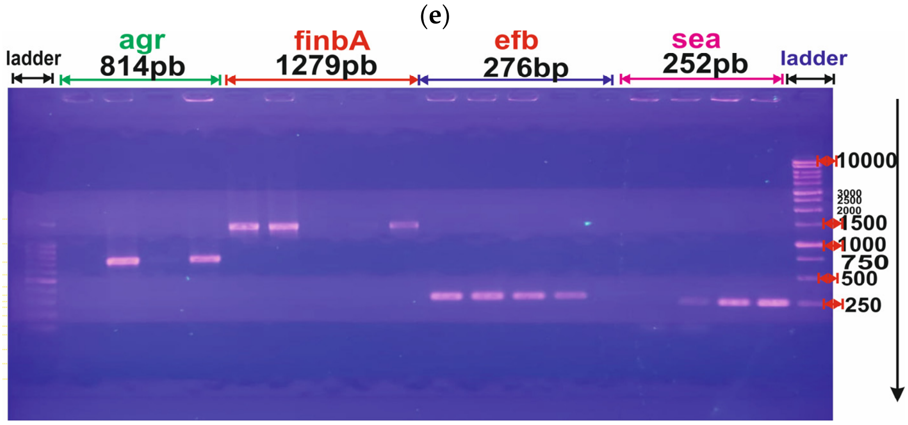

(**f**)

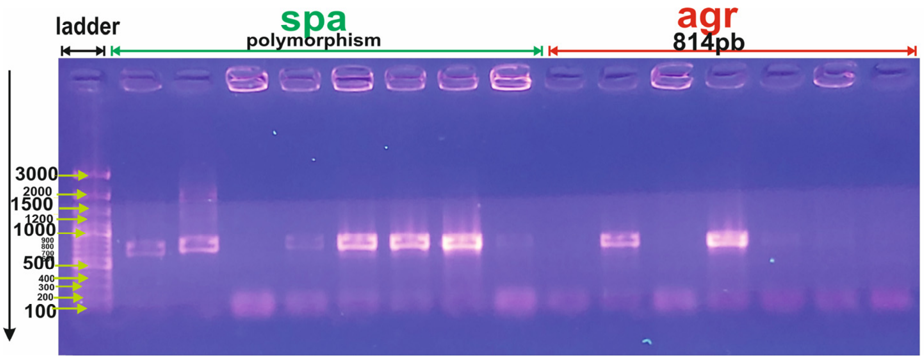

(**g**)

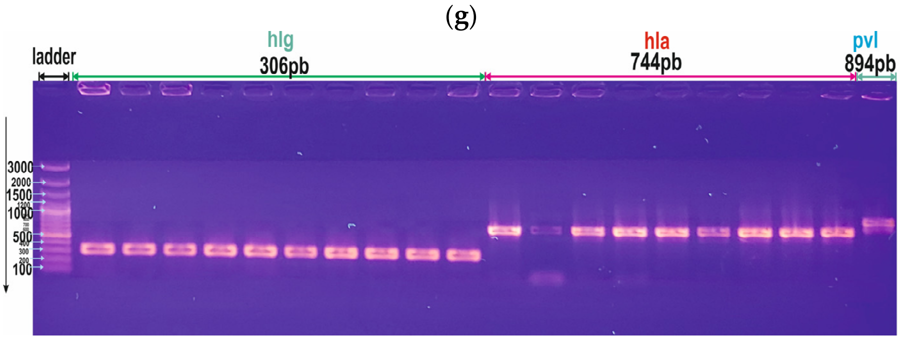

(h)

Figure S2. Cont.

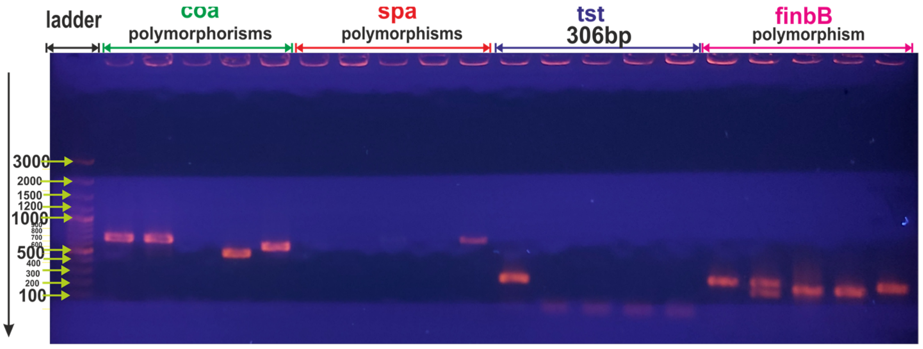

(**i**)

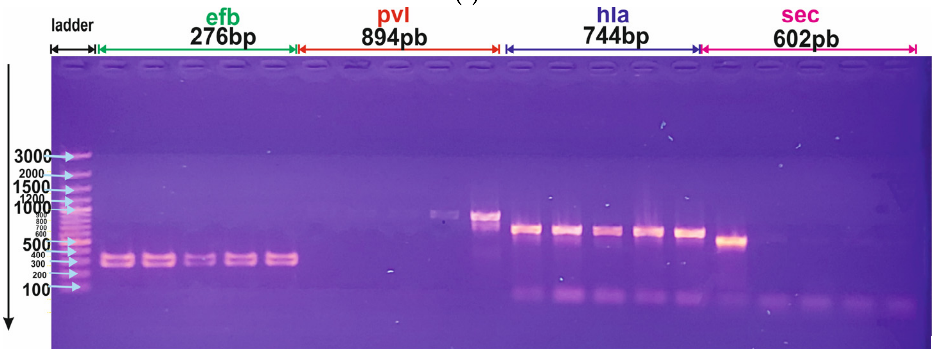

(j)

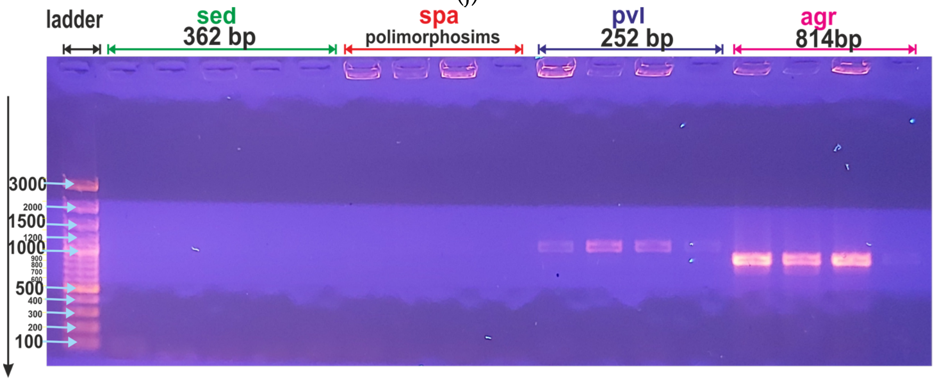

(**k**)

Figure S2. Agarose gel electrophoresis of the amplified adhesion-associated genes (*clfA, fnbA, fnbB, sdrC, sdrD,* and *spa*), enterotoxin A, B and C genes (sea, seb, sec) and other exotoxin genes (*hla, hlg, pvl* and *tst),* and others (*coa, efb, icaA, nuc* and *agr*) virulence genes. 25 µl of the PCR product was separated in 1% agarose containing ethidium bromide solution (1 µg/ml), and visualized using Gel documentation system. Lane ladder: 100bp; Other lanes: S. aureus strain for different virulence genes.

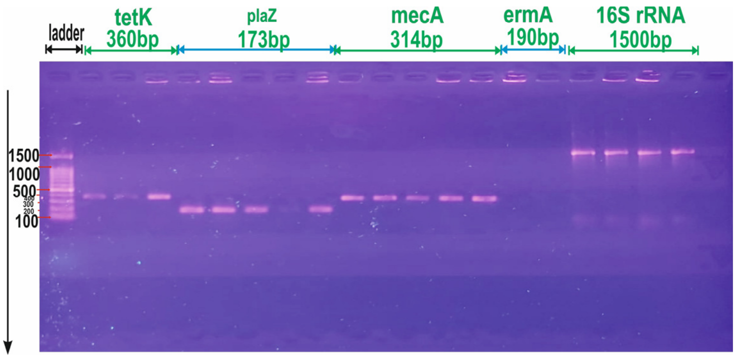

(**a**)

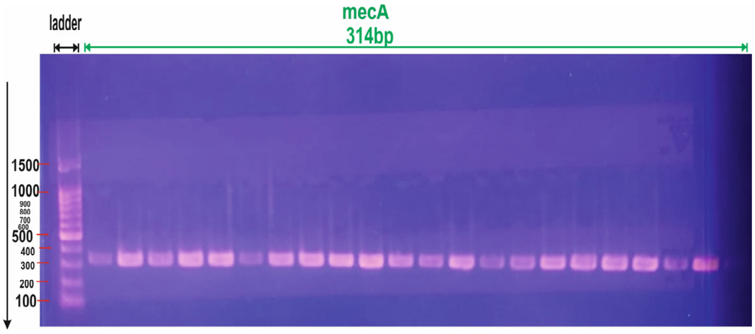

(**b**)

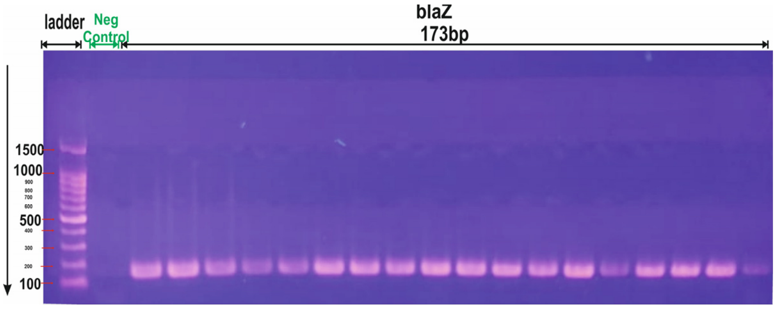

(**c**)

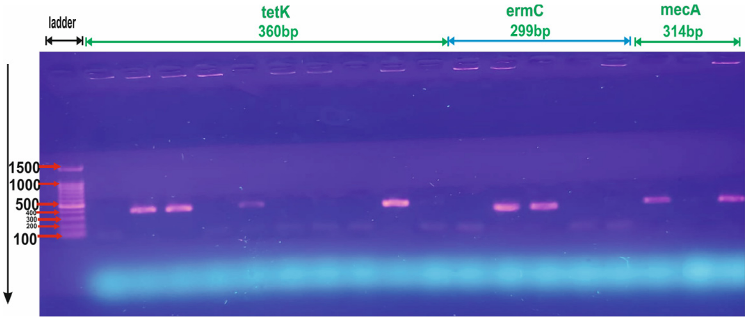

(d)

Figure S3. Agarose gel electrophoresis of the amplified *tetK, plaZ, mecA, ermA* and *ermC* antibiotics resistance genes. 25 µl of the PCR product was separated in 1% agarose containing ethidium bromide solution (1 µg/ml), and visualized using Gel documentation system. M: 100bp ladder; other lanes: S. aureus strains.
